# Human Placenta-Derived Mesenchymal Stem Cells Reduce Mortality and Hematoma Size in a Rat Intracerebral Hemorrhage Model in an Acute Phase

**DOI:** 10.1155/2018/1658195

**Published:** 2018-05-02

**Authors:** Bo Young Choi, Ok Joon Kim, Sae-Hong Min, Jeong Hyun Jeong, Sang Won Suh, Tae Nyoung Chung

**Affiliations:** ^1^Department of Physiology, College of Medicine, Hallym University, Hallymdaehak-gil, Chuncheon 24252, Republic of Korea; ^2^Department of Neurology, CHA University School of Medicine, 59 Yatap-Ro, Bundang-Gu, Seongnam 13496, Republic of Korea; ^3^Department of Emergency Medicine, CHA University School of Medicine, 59 Yatap-Ro, Bundang-Gu, Seongnam 13496, Republic of Korea

## Abstract

Intracerebral hemorrhage (ICH) is a critical disease, highly associated with mortality and morbidity. Several studies have demonstrated the beneficial effect of mesenchymal stem cells (MSCs) on ICH, mostly focused on their mid-to-long-term effect. Acute hematoma expansion is one of the most important prognostic factors of ICH. We hypothesized that MSCs would decrease mortality and hematoma size in acute ICH, based on the findings of a few recent researches reporting their effect on blood-brain barrier and endothelial integrity. Rat ICH models were made using bacterial collagenase. One hour after ICH induction, the rats were randomly divided into MSC-treated and control groups. Mortality, hematoma volume, ventricular enlargement, brain edema, and degenerating neuron count were compared at 24 hours after ICH induction. Expression of tight junction proteins (ZO-1, occludin) and coagulation factor VII mRNA was also compared. Mortality rate (50% versus 8.3%), hematoma size, ventricular size, hemispheric enlargement, and degenerating neuron count were significantly lower in the MSC-treated group (*p* = 0.034, 0.038, 0.001, 0.022, and <0.001, resp.), while the expression of ZO-1 and occludin was higher (*p* = 0.007 and 0.012). Administration of MSCs may prevent hematoma expansion in the hyperacute stage of ICH and decrease acute mortality by enhancing the endothelial integrity of cerebral vasculature.

## 1. Introduction

Nontraumatic intracerebral hemorrhage (ICH) is highly associated with mortality and morbidity, with a substantially worse prognosis than ischemic stroke [1, 2]. Moreover, it is highly associated with acute mortality; nearly 50% of patients die before 30 days after the onset of ICH, and half of them die within 48 hours [3–5]. Though various therapeutic approaches to overcome the extremely poor prognosis of ICH have been studied, including the administration of neuroprotective agents and exogenous coagulation factors, only symptomatic treatments are currently considered effective therapeutic options for ICH [2, 6, 7]. Stem cell therapy is currently regarded as one of the most promising strategies for the treatment of many incurable diseases and has shown neuroprotective effects on various neuronal injury and degenerative disease models. Various preclinical studies have also shown the beneficial neuroprotective effects of stem cell therapy for ICH via secretion of neurotrophic factors [8, 9].

In addition to loss of brain parenchymal tissue, early hematoma expansion is known to be an important prognostic factor of ICH that can predict mortality and poor functional outcome [1, 10, 11]. Various medical therapies to overcome poor prognosis due to hematoma expansion, including the administration of corticosteroids, glycerol, and mannitol, have been studied, but none of these have shown beneficial effects in clinical trials [12–14]. Recently, recombinant coagulation factor VII administration, an approach to improve the prognosis of acute ICH by limiting early hematoma growth via the factor's acute hemostatic effect, showed potential benefits in an early-phase clinical trial but failed to show a significant effect in a phase III clinical trial [15, 16]. Most studies of stem cell therapy for ICH have focused on neuronal death, functional outcome, and hematoma size in subacute-to-chronic stages of the disease but not on the prognosis of the acute-stage or early hematoma expansion [8, 9]. Results of recent studies showing that the administration of mesenchymal stem cells (MSCs) prevents blood-brain barrier (BBB) disruption and endothelial damage suggest that MSCs may improve the prognosis of ICH through the prevention of ongoing bleeding in the acute stage by intensification of the cerebral vasculature [17, 18].

We aimed to assess the effect of MSCs on ICH, specifically focusing on prognosis and hematoma size in the acute stage, and to suggest a possible mechanism. We hypothesized that the administration of MSCs would decrease mortality and hematoma size in the acute stage of ICH through a mechanism associated with the enhancement of cerebrovascular integrity.

## 2. Methods

### 2.1. Experimental Animals

Animal care protocol and experimental procedures were approved by the Institutional Animal Care and Use Committee of Hallym University (Protocol number Hallym 2013-126). All experiments were performed in accordance with relevant guidelines and regulations. Adult male Sprague-Dawley rats (250–350 g) were used in this study. Rats were housed in a regulated environment (22 ± 2°C, 55 ± 5% humidity, and 12 : 12-hour light : dark cycle with lights on at 8:00 am) and received a standard diet (Purina, Gyeonggi, Korea). Food and water were accessible ad libitum.

### 2.2. Intracerebral Hemorrhage (ICH) Model

**Figure 1:**
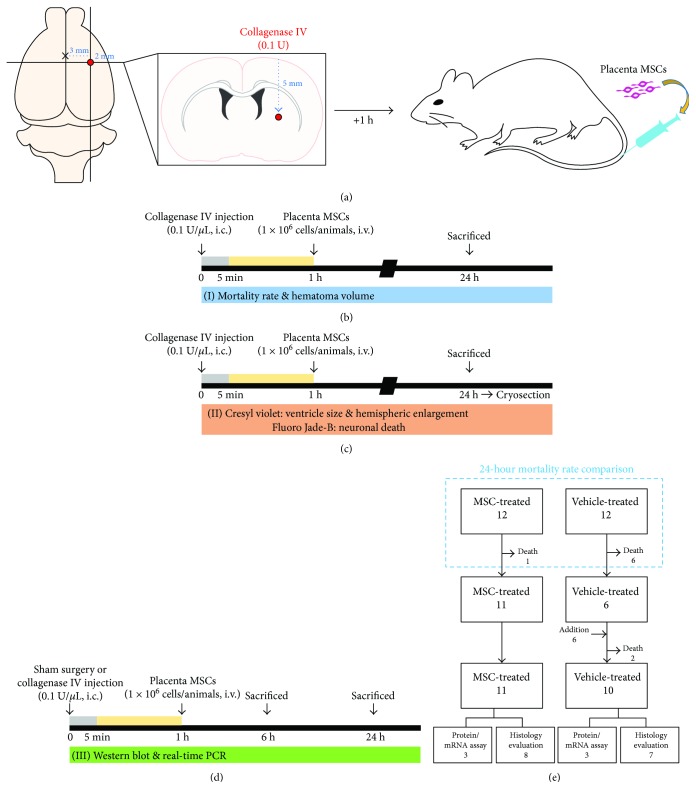
Conceptual illustrations of the experimental protocol. (a) Schematic diagrams of a rat intracranial hemorrhage (ICH) model with the administration of human placenta-derived mesenchymal stem cells (PD-MSCs). ICH was induced by the infusion of bacterial collagenase type IV (0.1 U, 1 *μ*L) into the striatum. PD-MSCs (1 × 10^6^ cells) were injected slowly via the tail vein at 1 hour after ICH. (b–d) Brief timeline of the experimental procedures which represent all the animal cohorts used and analyses performed: (b) mortality rate and hematoma volume; (c) ventricle size, hemispheric enlargement, and neuronal death; and (d) Western blot and real-time PCR. (e) Flow diagram which described how rats were used in a stepwise fashion.

To reproduce ICH with ongoing bleeding, we injected bacterial collagenase intrastriatally, as previously described [19]. Rats were deeply anesthetized with isoflurane (3% for induction, 1-2% for maintenance) in a 70 : 30 mixture of nitrous oxide and oxygen using an isoflurane vaporizer (VetEquip Inc., Livermore, CA) and were placed in a stereotaxic frame (Kopf Instruments, Tujunga, CA). A burr hole was made, and a 30-gauge needle was inserted through the burr hole into the striatum (coordinates: 0.2 mm posterior, 5.0 mm ventral, and 3.0 mm lateral to the bregma). We then injected collagenase type IV (0.1 U, 1 *μ*L) for 5 min (Figure 1(a)). After placement for another 4 min, the needle was removed slowly. The burr hole was sealed with bone wax. Following suture of the skin incision, anesthetics were discontinued. When rats showed spontaneous respiration, they were returned to a recovery room maintained at 37°C. Core temperature was kept at 36.5–37.5°C with a homoeothermic blanket control (Harvard Apparatus, Holliston, MA). Sham-operated rats received the same neck skin incision under isoflurane anesthesia, but they were administered 1 *μ*L sterile saline into the right striatum.

### 2.3. MSC Preparation and Experimental Procedures

Human placenta-derived mesenchymal stem cells (PD-MSCs) were isolated and characterized as previously described [20]. Placenta tissue was obtained with informed consent from healthy mother donors, under the approval of the institutional review board of CHA Bundang Medical Center. The chorioamniotic membrane was separated from the placenta, and the amnion and innermost membrane from the chorion and decidua were removed. The cells of the chorionic plate side were removed from the membrane, the remainder of which was dissected and minced. The minced tissue was enzymatically digested by a mixture of trypsin, DNase I, and collagenase IV at 37°C for 30 min under shaking conditions. The harvested cells were cultured in T25 flasks in MEM-*α* GlutaMAX supplemented with 10% fetal bovine serum, 25 ng/mL FGF4 (R&D System, Minneapolis, MN), and 1 *μ*g/mL heparin at 37°C in an atmosphere of 5% CO_2_ and 3% O_2_. Fluorescence-activated cell sorting analysis was used to identify the phenotype of the cells. The expression of CD44, CD73, CD90, CD105, and human leukocyte antigen- (HLA-) ABC and the lack of CD45, CD34, CD31, and HLA-DR were assessed to confirm the MSC identity of the cells.

One hour after ICH induction, the animals were randomly assigned to two groups: the MSC-treated group, composed of those receiving a 500 *μ*L suspension of PD-MSCs (1 × 10^6^ cells) slowly for 5 min via the tail vein, and the vehicle-treated group, composed of those receiving the same volume of saline. The experimental procedures are summarized in Figure 1. Mortality rates at 24 hours after ICH induction were calculated, and surviving animals were sacrificed to acquire brain samples for analyses at the same time point. The same procedures were repeated to add more animals, as the sample size was insufficient for statistical analyses of histological evaluation in any of the groups because of mortalities.

### 2.4. Tissue Preparation

For the histological evaluation, rats were anesthetized by intraperitoneal injection of 1.5 g/kg urethane in sterile 0.9% NaCl at a volume of 0.01 mL/g body weight. A toe pinch was used to evaluate the effectiveness of anesthesia. Animals were transcardially perfused with 0.9% saline followed by 4% paraformaldehyde (PFA) in PBS. The brains were postfixed with 4% PFA in PBS for 1 hour and then immersed in 30% sucrose for cryoprotection. Thereafter, the entire brain was frozen and coronally sectioned with a cryostat microtome at 30 *μ*m thickness.

For the analysis of Western blot and real-time polymerase chain reaction (PCR), rats were anesthetized and perfused with cold PBS. The brains were immediately harvested.

### 2.5. Measurement of the Hematoma Volume

For the measurement of hematoma volume at 24 hours after ICH, rats were divided into two groups. These groups were composed of a vehicle-treated ICH group (*n* = 7) and MSC-treated ICH group (*n* = 8). The hematoma volume was quantified using coronal sections at 28 rostral-caudal levels that were spaced every 270 *μ*m from +2.04 mm to −5.52 mm relative to the bregma. The volume measurement was computed by summation of the areas multiplied by the interslice distance (270 *μ*m). Digital photographs of the serial slices were taken, and the percentage of hematoma volume [(hematoma volume/hemispheric brain volume) × 100] was measured using ImageJ (NIH, Bethesda, MA) [21]. This analysis was performed by an investigator blinded to the experimental cohort.

### 2.6. Ventricle Size and Hemispheric Enlargement Analysis

For the investigation of ventricle size and hemispheric enlargement at 24 hours after ICH, rats were divided into two groups. These groups were composed of a vehicle-treated ICH group (*n* = 7) and MSC-treated ICH group (*n* = 8). Brains were cut coronally with a cryostat microtome at 30 *μ*m thickness. Sections were stained with cresyl violet and visualized under a light microscope (Olympus upright microscope IX70, Olympus, Tokyo, Japan). The whole brain area and the area of the ventricle were measured using ImageJ, with the area of the ventricle expressed as a percentage of the total brain area [22–24]. As a previously described method [25], brain edema was measured using ImageJ software as the percentage of hemispheric enlargement, which was calculated by the following formula: [(ipsilateral hemisphere volume − contralateral hemisphere volume)/contralateral hemisphere volume] × 100 [25–27]. These analyses were performed by an observer blinded to the experimental cohort.

### 2.7. Detection of Neuronal Death

Neuronal death was evaluated by Fluoro-Jade B (FJB, Histo-Chem, Jefferson, AR) staining 24 hours after ICH [28]. The sections were rinsed in PBS and mounted onto gelatin-coated slides and then dried on a slide warmer. The slides were immersed in 100% ethanol for 3 min, followed by 70% ethanol for 1 min and distilled water (DW) for 1 min. The slides were then transferred to 0.06% potassium permanganate for 15 min and gently agitated. After rinsing in DW for 1 min, the slides were incubated for 30 min in 0.001% FJB, freshly prepared by adding 20 mL of a 0.01% FJB solution to 180 mL of 0.1% acetic acid, with gentle shaking in the dark. After rinsing for 1 min in each of three changes of DW, the slides were dried, dehydrated in xylene, and coverslipped with DPX (Sigma-Aldrich Co., St. Louis, MO). To quantify neuronal death in the perihematomal region after ICH, rats were divided into two groups. These groups were composed of a vehicle-treated ICH group (*n* = 7) and MSC-treated ICH group (*n* = 8). Sections were collected at +1.2 mm to −1.2 mm from the bregma according to the coordinates of Slotnick and Leonard [29], and seven coronal sections were analyzed from each animal using a microscope with a 20x objective. An observer masked to the treatment condition counted the number of FJB-positive (+) neurons by sampling an area of 4.72 × 4.72 mm^2^ immediately adjacent to the hematoma in 3 regions of interest (ROIs) from the ipsilateral hemisphere after ICH. The number of FJB-positive cells from 21 randomly selected locations per mouse (3 fields per section × 7 sections per mouse) in the perihematomal region was averaged and expressed as FJB-positive cells per square millimeter.

### 2.8. Western Blot

Rats were perfused with cold PBS at 6 or 24 hours after ICH or sham operation for the analysis of the expression of tight junction proteins using Western blot. A 2 mm coronal section (+0.8 mm to −1.2 mm relative to the bregma) from the ipsilateral hemisphere was homogenized in ice-cold RIPA buffer (Thermo Fisher Scientific, Waltham, MA). Total protein concentration was measured by the BCA Protein Assay Kit (Thermo Fisher Scientific, Waltham, MA). Then, 40 *μ*g of protein from each sample was subjected to SDS-PAGE and transferred to a PVDF membrane (GE Healthcare Bio-Sciences, Pittsburgh, PA). Membranes were probed with the following primary antibodies: rabbit anti-occludin (Thermo Fisher Scientific, Waltham, MA), rabbit anti-zonula occludens-1 (ZO-1) (Abcam, Cambridge, MA), and mouse anti-*β*-actin (Santa Cruz Biotechnology Inc., Dallas, TX). *β*-Actin was used as an internal loading control. The secondary antibodies were all from GeneTex. Western blot was performed with an ECL Detection Kit (Bio-Rad, Hercules, CA). The relative band density of each sample was analyzed using ImageJ. These analyses were performed by an observer blinded to the experimental cohort.

### 2.9. Real-Time PCR

For the analysis of the expression of coagulation factor VII (F7) mRNA at 6 or 24 hours after ICH or sham operation, we performed real-time polymerase chain reaction (PCR). Total RNA was isolated from ipsilateral regional brain tissue using NucleoZOL (Macherey-Nagel, Düren, Germany) following the manufacturer's instruction. cDNA was synthesized using the PrimeScript™ 1st-strand cDNA Synthesis Kit (Takara Bio, Shiga, Japan). mRNA was quantified using iQ™ SYBR® Green Supermix (Bio-Rad) with the CFX Connect™ Real-Time PCR Detection System (Bio-Rad). Thermal cycling parameters were determined from the manufacturer's instruction (2 min at 95°C and 40 cycles of 95°C for 10 s, 60°C for 10 s, and 72°C for 30 s). The following primers were used: rat F7-specific primers (forward, GCT TCT GCC CCC TAG ACT TT; reverse, CCG CAT GGG TAC TCA ACT TT) and rat GAPDH-specific primers (forward, ACC ACA GTC CAT GCC ATC AC; reverse, TCC ACC ACC CTG TTG CTG TA). The relative band density of each sample was analyzed using ImageJ. These analyses were performed by an observer blinded to the experimental cohort.

### 2.10. Statistical Analysis

Comparisons between the MSC-treated and vehicle-treated groups were conducted using the *t*-test, except for the comparison of mortalities, which used Fisher's exact test. Data are presented as the mean ± standard error of the mean (SEM), and differences were considered significant at *p* < 0.05. IBM SPSS statistics 24.0 (IBM, Armonk, NY) was used for statistical calculation. A sample size of 20 was calculated to detect a significant difference in the proportion of deaths at 24 hours after ICH induction between the MSC-treated and vehicle-treated groups (power = 0.8), using G∗Power 3.1 (Heinrich-Heine Universität, Düsseldorf, Germany) [30].

## 3. Results

First, 24 rats were assigned to two groups (12 each). Six rats in the vehicle-treated group died before 24 hours had passed, while one rat in the MSC-treated group died before 24 hours. Six more rats were enrolled and assigned to the vehicle-treated group, and among them, two rats died within 24 hours. Finally, seven rats in the vehicle-treated group and eight rats in the MSC-treated group were included in the histologic analyses, and three rats in each group were enrolled for Western blot and real-time PCR (Figure 1(e)).

### 3.1. PD-MSCs Decreased Mortality Rate and Hematoma Volume in an Acute Stage of ICH

**Figure 2:**
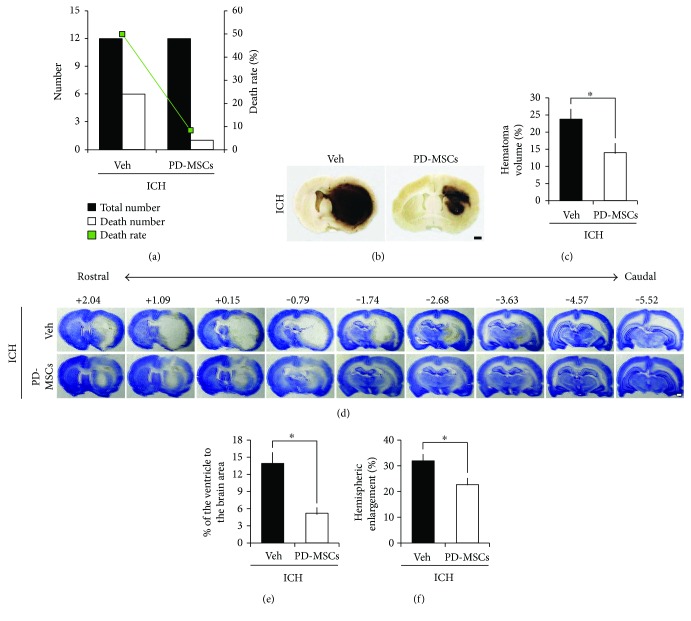
Effect of human placenta-derived mesenchymal stem cell (PD-MSC) administration on mortality, hematoma volume, ventricular enlargement, and brain edema of the rats at 24 hours after the induction of intracranial hemorrhage (ICH). (a) Mortalities in each group at 24 hours after ICH (*n* = 12 from each group). (b) Digital photographs showing location of a core hemorrhagic region at 0.2 mm from the bregma. Scale bar = 1 mm. (c) The bar graphs represent the hematoma volume of the vehicle-treated and the PD-MSC-treated groups at 24 hours after ICH induction. The volume of hematoma is expressed as the proportion in the total brain area (%). Data are mean + SEM; *n* = 7-8 from each group (ICH-Veh, *n* = 7; ICH-MSC, *n* = 8), ^∗^*p* < 0.05. (d) Representative images of cresyl violet staining depicting a coronal whole-brain section at rostral-caudal levels from +2.04 to −5.52 from the bregma. Scale bar = 1 mm. (e) The bar graphs represent the ventricular size of the vehicle-treated and the PD-MSC-treated groups at 24 hours after ICH induction. The size of the lateral ventricle is expressed as the proportion in the total brain area (%). Data are mean + SEM; *n* = 7-8 from each group (ICH-Veh, *n* = 7; ICH-MSC, *n* = 8), ^∗^*p* < 0.05. (f) The bar graphs represent the degree of the hemispheric enlargement of the vehicle-treated and the PD-MSC-treated groups at 24 hours after ICH induction. The hemispheric enlargement is expressed as the percentage of increase in hemispheric size compared with that of the contralateral hemisphere. Data are mean + SEM; *n* = 7-8 from each group (ICH-Veh, *n* = 7; ICH-MSC, *n* = 8), ^∗^*p* < 0.05.

Administration of PD-MSCs significantly reduced the mortality rate from 50% (6 of 12 rats) in the vehicle-treated group to 8.3% (1 of 12 rats) in the MSC-treated group (*p* = 0.034, Figure 2(a)). The most important predictor of early death is the size of the hematoma after brain injury, followed by ICH. To assess whether the reduction in mortality rate in the MSC-treated group correlated with reduced hematoma size, we examined the hematoma size at 24 hours following ICH. Hematoma size was significantly smaller in the MSC-treated group (13.98 ± 2.59%) than in the vehicle-treated group (23.73 ± 2.81%, *p* = 0.038; Figures 2(b) and 2(c)). These results suggest that administration of PD-MSCs decreases the mortality rate and the hematoma size in the acute stage of ICH.

### 3.2. PD-MSCs Reduced Ventricular Enlargement and Brain Edema after ICH

To determine whether administration of PD-MSCs decreased ventricular enlargement and brain edema after ICH, we measured changes in the lateral ventricle and hemispheric enlargement, respectively. The ventricular size and hemispheric volume were unchanged at the designated time point in animals that underwent sham surgery (data not shown). At 24 hours after ICH, the vehicle-treated rats exhibited larger ventricle size than the sham-operated group, and the ventricle size was reduced in the MSC-treated group compared to the vehicle-treated group (13.89 ± 1.87% versus 5.2 ± 0.91%, *p* = 0.001; Figures 2(d) and 2(e)). In addition, hemispheric enlargement was significantly smaller in the MSC-treated group (22.64 ± 2.55%) than in the vehicle-treated group (31.90 ± 2.44%, *p* = 0.022; Figures 2(d) and 2(f)). These results indicate that PD-MSC treatment attenuates ICH-induced brain edema formation and hydrocephalus.

### 3.3. PD-MSCs Reduced Neuronal Death after ICH

**Figure 3:**
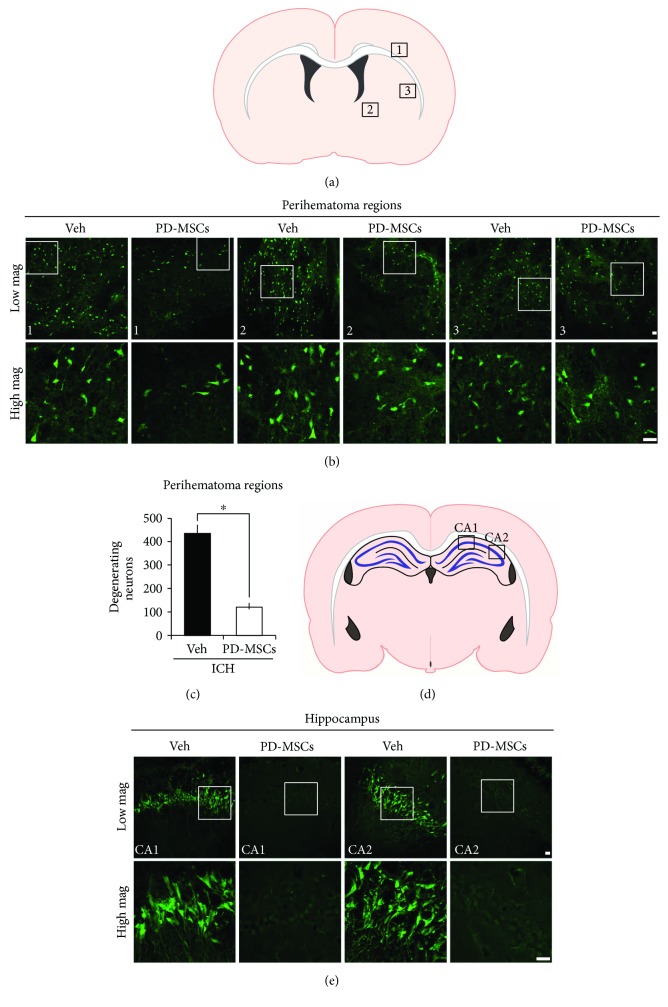
Human placenta-derived mesenchymal stem cells (PD-MSCs) reduced neuronal death in the brains of rats subjected to intracranial hemorrhage (ICH). (a) The location of core hemorrhagic regions at 0.2 mm from the bregma. (b) Fluorescence images reveal the degenerating neurons in the perihematomal region at 24 hours after ICH. Degenerating neurons are detected by Fluoro-Jade B (FJB) staining (green). Scale bar = 20 *μ*m. (c) The bar graphs represent the count of FJB-positive neurons in the perihematomal region from the vehicle-treated and the PD-MSC-treated groups at 24 hours after ICH induction. Data are mean + SEM; *n* = 7 − 8 from each group (ICH-Veh, *n* = 7; ICH-MSC, *n* = 8), ^∗^*p* < 0.05. (d) The location of hippocampal regions at −3.6 mm from the bregma. (e) Fluorescence images reveal the degenerating neurons only in the hippocampal CA1 and CA2 region of the vehicle-treated group at 24 hours after ICH. Scale bar = 20 *μ*m.

To determine the neuroprotective effect of PD-MSCs on collagenase-induced ICH, we performed FJB staining to detect degenerating neurons. The MSC-treated group had significantly fewer FJB-positive cells in the perihematomal region 24 hours after ICH than the vehicle-treated group (433.22 ± 34.17 versus 120.36 ± 15.22 cells/field, *p* < 0.001; Figures 3(a)–3(c)). Moreover, contrary to the vehicle-treated group, no FJB-positive cells were observed in the ipsilateral hippocampus of the MSC-treated group at 24 hours after ICH (Figures 3(d) and 3(e)). FJB-positive cells were not observed in the contralateral hemisphere. These data suggest that administration of PD-MSCs effectively reduces neuronal death after collagenase-induced ICH injury.

### 3.4. PD-MSCs Increased the Expression of the Tight Junction Proteins after ICH

**Figure 4:**
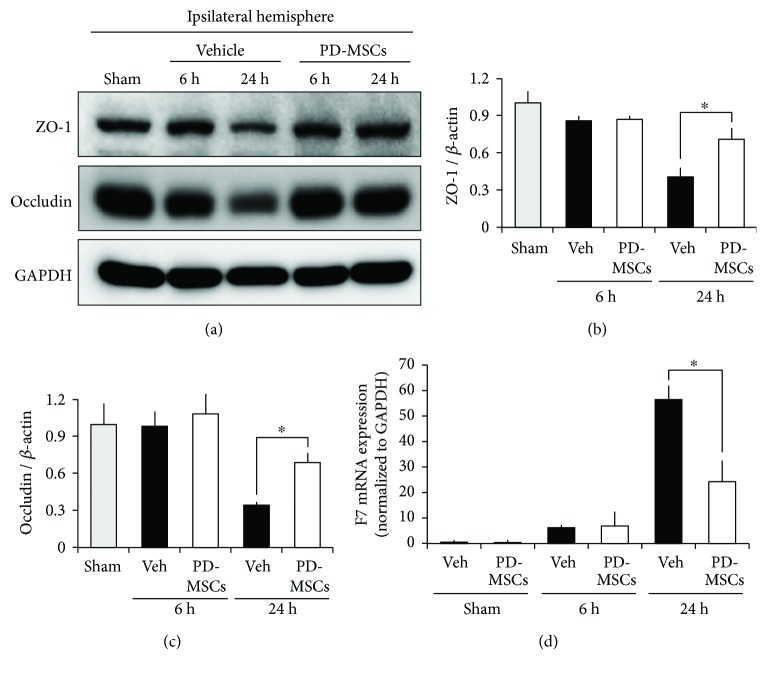
Human placenta-derived mesenchymal stem cells (PD-MSCs) enhanced the expression of tight junction proteins at 24 hours after ICH induction but did not affect the expression of coagulation factor VII mRNA. (a) Results of Western blot of ZO-1 and occludin at 6 and 24 hours after ICH induction. Bar graphs indicate the level of ZO-1 (b) and occludin (c) expression measured by the densitometric analysis of the bands. *β*-Actin was used as a loading control. Data are mean + SEM; *n* = 3 from each group, ^∗^*p* < 0.05. (d) Real-time PCR analysis of coagulation factor VII (F7) at 6 and 24 hours after ICH induction, and that at 24 hours after sham operation. The level of F7 mRNA was normalized to that of GAPDH mRNA. Data are mean + SEM; *n* = 3 from each group, ^∗^*p* < 0.05.

We investigated the expression of tight junction proteins at 6 and 24 hours after ICH to assess changes in microvascular integrity using Western blot. The level of expression of ZO-1 and occludin was significantly higher in the MSC-treated group than in the vehicle-treated group at 24 hours after ICH induction (*p* = 0.007 and 0.012), but not at 6 hours after ICH induction (*p* = 0.744 and 0.558, Figures 4(a)–4(c)). These results suggest that PD-MSCs block the leakage of blood components from ruptured vessels to brain parenchyma after ICH, indicating an enhancement of tight junction barrier function.

### 3.5. PD-MSCs Did Not Affect the Level of F7 mRNA Expression

We investigated the expression of F7 mRNA at 6 and 24 hours after ICH or sham operation. The level of F7 mRNA expression was significantly higher in the vehicle-treated group than in the MSC-treated group at 24 hours after ICH induction (*p* = 0.003) but not at 6 hours after ICH induction (*p* = 0.861, Figure 4(d)). However, there was no significant difference in the level of F7 mRNA expression between the vehicle-treated and MSC-treated groups at 24 hours after sham operation (*p* = 0.963, Figure 4(d)).

## 4. Discussion

In this study, we found that PD-MSC administration decreased the mortality of ICH in the acute stage by suppressing hematoma expansion and by various neuroprotective effects, including the amelioration of hydrocephalus, perihematomal neuronal death, and brain edema. The present study also showed that administration of PD-MSCs increased the expression of tight junction proteins associated with the enhancement of cerebrovascular integrity. These results suggest that PD-MSCs may have a high therapeutic potential for treating acute-phase ICH.

Here, we showed that the systemic administration of PD-MSCs decreased mortality, hematoma size, and brain edema/hydrocephalus at 24 hours after ICH induction. This suggests that stem cell therapy may be useful not only as a treatment option for functional recovery after ICH because of its mid-to-long-term neuroprotective, neurotrophic, and regenerative effects but also as a treatment option for decreasing acute-stage mortality and severe complications of ICH. Recently, many trials have examined the use of MSCs in the field of acute/intensive care medicine because of their effects of reducing inflammation and preventing systemic ischemia/reperfusion injury [31]. Therefore, the results of our study also suggest that administration of MSCs may be used as a complementary therapeutic option to the conventional therapy for acute-stage ICH including surgical intervention, which may significantly improve prognosis.

The hematoma area reduction effect of stem cell therapy for ICH is well known from the results of various preclinical studies [32–36]. Most of these studies focused on the mid-to-long-term replacement of lost brain tissue through the neurotrophic and neuronal regeneration effects of administered stem cells. In contrast, our study showed that the administration of PD-MSCs had a strong effect of hematoma size reduction even in the acute stage of ICH. This suggests that administered stem cells may also directly affect suppression of hematoma expansion, considering that there was not enough time for regeneration and replacement of lost tissue. The results of the Western blot, which showed significantly higher expression of tight junction proteins in the MSC-treated group at 24 hours after ICH induction, suggest that suppression of acute hematoma expansion may be due to PD-MSC-mediated enhancement of the endovascular integrity of brain microvasculature in a relatively short time. This finding is consistent with that of previous research, which shows that MSC administration decreases blood-brain barrier permeability and endothelial damage and increases the expression of tight junction proteins after ICH [17, 18, 37]. In most cases of ICH, hematoma expansion occurs within 24 hours after the onset regardless of its extent, implying that there may be an active bleeding process in the hyperacute phase of ICH [1, 38]. Thus, our results showing a significant decrease in hematoma size and increase in vascular integrity at the time point of 24 hours after ICH induction suggest that MSC administration in the early stage of ICH may effectively attenuate this active bleeding process.

Because hydrocephalus and cerebral edema, which reflect and also cause an increase in intracranial pressure (ICP), are the major complications of ICH, most therapeutic strategies for the acute stage of ICH focus on preventing and treating these complications [2, 6]. We showed a significant decrease in ventricular size and hemispheric enlargement in the MSC-treated group compared with the vehicle-treated group, suggesting that the amelioration of these serious complications of ICH may be an important mechanism underlying the effects of PD-MSCs on acute mortality from ICH. In addition, the significant decrease in neuronal death in the MSC-treated group, observed by FJB immunostaining, suggests that direct neuroprotective effects of PD-MSCs may also contribute to improved prognosis. In particular, the absence of degenerating neurons in the hippocampus of the MSC-treated group, compared to the presence of degenerating neurons in the vehicle-treated group, implies that MSCs may also ameliorate global ischemic injury caused by increased ICP. However, further study may be necessary to elucidate whether this result is a mere consequence of the decrease in hematoma expansion and ICP or due to the direct neuroprotective effects of MSCs.

A few recent studies reported that MSCs have procoagulant features [39, 40]. Notably, one study showed that MSCs contribute to the production of endogenous coagulation factor VIII [41]. Thus, we aimed to determine whether MSCs would increase the production of F7, which has been used as an acute hemostatic therapy for ICH. However, we found no significant difference in the expression of F7 mRNA between the MSC-treated and the vehicle-treated sham-operated animals, indicating that PD-MSCs do not cause an increase in endogenous F7 in the ICH model. Rather, we showed a significantly higher level of F7 mRNA expression in the vehicle-treated group than in the MSC-treated group at 24 hours after ICH induction. This increase in mRNA expression may be a response to the increased consumption of coagulation factor due to ongoing bleeding, which was more pronounced in the vehicle-treated group. Additional studies are needed to determine whether the effect of MSCs on acute hematoma expansion is related to their procoagulant properties or to other factors in the coagulation system.

Our study has a few limitations. First, we used a collagenase injection model to reproduce ICH with acute hematoma expansion, producing a pathophysiology quite different from the real disease, which is usually caused by mechanical tension due to hypertension or aneurysm. However, the pronounced effects shown in this model, which involve a strong trend of ongoing bleeding and hematoma expansion due to the continuous action of injected collagenase, suggest that MSCs may demonstrate therapeutic efficacy in a real clinical setting. Moreover, this model is known to generate consistent and predictable ICH and provides the best imitation of the bleeding-rebleeding phenomenon of the real human condition [42]. Second, we did not assess the multiorgan effect of systemically administered MSCs. Considering the systemic effects of MSCs on inflammation and ischemia/reperfusion injury, the effects on other vital organs might also contribute to the decreased acute mortality in our results. Third, our quantification methods for measuring hemispheric and ventricular enlargement cannot exclusively reflect overall brain edema. Hence, the effect of MSCs on ICH-induced brain edema formation and hydrocephalus might be a consequence of smaller hemorrhage volume. Further study may be necessary to examine this possibility in depth.

## 5. Conclusions

The administration of MSCs may prevent hematoma expansion in the hyperacute stage of ICH and decrease acute mortality by enhancing the endothelial integrity of cerebral vasculature, in addition to exerting their neuroprotective and neurotrophic effects as shown in previous studies.
